# Placental pathology in perinatal asphyxia: a case–control study

**DOI:** 10.3389/fcdhc.2023.1186362

**Published:** 2023-09-18

**Authors:** Silvia Alongi, Laura Lambicchi, Francesca Moltrasio, Valentina Alice Botto, Davide Paolo Bernasconi, Maria Serena Cuttin, Giuseppe Paterlini, Silvia Malguzzi, Anna Locatelli

**Affiliations:** ^1^ Department of Obstetrics and Gynecology, San Gerardo Hospital, University of Milano-Bicocca, Monza, MB, Italy; ^2^ Department of Obstetrics and Gynecology, Fondazione MONZA e BRIANZA per il BAMBINO e la sua MAMMA (MBBM), San Gerardo Hospital, Monza, MB, Italy; ^3^ Department of Pathology, Desio Hospital, Azienda Socio Sanitaria Territoriale (ASST) Brianza, Desio, MB, Italy; ^4^ School of Medicine and Surgery, University of Milano-Bicocca, Monza, MB, Italy; ^5^ Bicocca Bioinformatics Biostatistics and Bioimaging Centre – B4, School of Medicine and Surgery, University of Milano-Bicocca, Monza, MB, Italy; ^6^ Department of Pathology, Vimercate Hospital, Azienda Socio Sanitaria Territoriale (ASST) Brianza, Vimercate, MB, Italy; ^7^ Neonatal Intensive Care Unit, Fondazione MONZA e BRIANZA per il BAMBINO e la sua MAMMA (MBBM), San Gerardo Hospital, Monza, MB, Italy; ^8^ Obstetrics, Fondazione Istituto di Ricovero e Cura a Carattere Scientifico (IRCCS) San Gerardo dei Tintori, Monza, Italy

**Keywords:** placenta, perinatal asphyxia, neonatal encephalopathy, meconium, malperfusion

## Abstract

**Introduction:**

Placentas of term infants with birth asphyxia are reported to have more lesion such as maternal vascular malperfusion (MVM), fetal vascular malperfusion (FVM) and chorioamnionitis with fetal response (FIR) than those of term infants without birth asphyxia. We compared the placental pathology of asphyxiated newborns, including those who developed hypoxic-ischemic encephalopathy (HIE), with non-asphyxiated controls.

**Methods:**

We conducted a retrospective case–control study of placentas from neonates with a gestational age ≥ 35 weeks, a birthweight ≥ 1,800 g, and no malformations. Cases were asphyxiated newborns (defined as those with an umbilical artery pH ≤ 7.0 or base excess ≤ −12 mMol, 10-minute Apgar score ≤ 5, or the need for resuscitation lasting >10 min) from a previous cohort, with (*n*=32) and without (*n*=173) diagnosis of HIE. Controls were non-asphyxiated newborns from low-risk l (*n*= 50) or high-risk (*n*= 68) pregnancies. Placentas were analyzed according to the Amsterdam Placental Workshop Group Consensus Statement 2014.

**Results:**

Cases had a higher prevalence of nulliparity, BMI>25, thick meconium, abnormal fetal heart monitoring, and acute intrapartum events than controls (*p*<0.001). MVM and FVM were more frequent among non-asphyxiated than asphyxiated newborns (*p*<0.001). There was no significant difference in inflammatory lesions or abnormal umbilical insertion site. Histologic meconium-associated changes (MAC) were observed in asphyxiated newborns only (*p*= 0.039).

**Discussion:**

Our results confirm the role of antepartum and intrapartum risk factors in neonatal asphyxia and HIE. No association between neonatal asphyxia and placental lesions was found, except for in the case of MAC. The association between clinical and placental data is crucial to understanding and possibly preventing perinatal asphyxia in subsequent pregnancies.

## 1 Introduction

Perinatal asphyxia is a condition of impaired gas exchange, which causes the fetus to suffer progressive hypoxia (1). One of its most serious consequences, neonatal hypoxic-ischemic encephalopathy (HIE), has an estimated incidence of 1.5 per 1,000 live births and can cause cerebral palsy, death, epilepsy, and cognitive impairment (2). There are many prenatal and perinatal risk factors implicated in the genesis of perinatal asphyxia and HIE, including nulliparity, obesity, fetal growth restriction (FGR), hypertensive disorders, hyperpyrexia during labor, and meconium-stained amniotic fluid (3–5).

Acute injuries occurring during labor, such as placental abruption and other sentinel events, have been demonstrated to be causes of perinatal asphyxia (6–9). However, chronic factors leading to an underperforming placenta can also cause remote antenatal damage (10), making the fetus more vulnerable to suffering hypoxia during labor. Furthermore, some conditions, such as hypertensive disease and diabetes, are associated with worse neonatal outcomes and abnormal placental patterns (11–15). Histopathologic studies on this topic have shown, albeit inconsistently, that the placentas of late preterm and term newborns with birth asphyxia contain elements of maternal vascular malperfusion, fetal vascular malperfusion, and chorioamnionitis with a fetal response (16), the latter two having been linked to a worse outcome and development of HIE (17, 18).

Given this lack of a strong correlation between specific placental pathology patterns and asphyxia, our first aim was to describe placental lesions present in cases of HIE and of asphyxia without short-term neonatal outcomes.

In addition, the literature describes specific placental lesions for some maternal and obstetric pathological conditions. Asphyxia can be preceded by both antepartum and intrapartum risk conditions. Thus, our second aim was to compare the placental pathology of newborns who developed perinatal asphyxia (with or without HIE) with those of physiological or high-risk pregnancies not complicated by perinatal asphyxia, in order to identify if and how placental analysis, according to current criteria, is able to differentiate between these groups.

## 2 Materials and methods

We conducted a retrospective case–control study on late preterm (≥ 35 gestational weeks) and term neonates. Cases were asphyxiated newborns without HIE (group A, *n*=173) and asphyxiated newborns with a diagnosis of HIE (group B, *n*=32) with placenta pathology available. Controls were non-asphyxiated newborns from physiological (group C, *n*=50) or high-risk pregnancies (group D, *n*=68).

The cases (groups A and B) were obtained from a previous cohort study conducted between July 2014 and June 2016 (7) in a network of four hospitals: one central university hospital with a neonatal intensive care unit that offers therapeutic hypothermia and three peripheral centers classed as level 1 community hospitals. Placental pathology was available in 68% of 253 asphyxiated newborns without diagnosis of HIE (*n*=173) and 81% of 16 asphyxiated newborns with diagnosis of HIE (*n*=13) collected in the cohort study. Subsequently, group B was supplemented with all cases of newborns with HIE born in the same network of hospitals until December 2019 with placental pathology available, reaching a total number of 32 cases of HIE.

The controls (groups C and D) were all subjects with placenta pathology available from a cohort collected prospectively from March to June 2020 in the same hospitals. The size of the control groups was considered adequate in relation to the size of the cases.

The inclusion criteria for all groups were as follows: gestational age ≥ 35 weeks, birthweight ≥ 1800 g, and no severe malformations.

Neonatal asphyxia was defined as a UA (umbilical artery) pH ≤ 7.0 or base excess ≤ −12 mMol or within 1 hour, a 10-minute Apgar score ≤ 5, or the need for resuscitation lasting > 10 minutes (19). In the first 6 hours of life asphyxiated neonates were monitored using the Thomson Score (TS), a numeric scoring system for the assessment of hypoxic ischemic encephalopathy consisting of a clinical assessment of nine signs (tone, level of consciousness, fits, posture, Moro reflex, palmar grasp, sucking reflex, respiratory pattern, and fontanel tension) (20). Each sign was scored from 0 to 3; if TS was ≥ 5, the neonate was transferred to the central University Hospital for further surveillance and if TS was ≥ 7, hypothermia was indicated. Encephalopathy was classified according to the Sarnat & Sarnat classification (21).

The inclusion criteria for group C were the absence of asphyxia at birth and of any antepartum or intrapartum risk factor.

The inclusion criteria for group D were the absence of asphyxia at birth and presence of at least one antepartum or intrapartum risk factor. We considered antepartum risk factors to be nulliparity, maternal age > 40 years, maternal BMI > 30 kg/m2 before pregnancy, oligohydramnios, polyhydramnios, FGR, induction of labor, maternal disease, and previous cesarean section. Intrapartum risk factors were sentinel events (fetal bradycardia, placental abruption, uterus rupture, shoulder dystocia, cord prolapse, and amniotic fluid embolism), thick meconium during labor, fever, and tachysystole.

Data regarding maternal history, pregnancy complications, fetal heart rate (FHR) monitoring, labor, and delivery were collected. FHR monitoring was classified according to the ACOG classification (22). Data recorded included birth weight, UA pH, UA BE, and UA lactate. In asphyxiated newborns, the need for ventilation by mask or endotracheal tube during initial stabilization, the need for cardiac massage, and biochemical and instrumental data were recorded (data available on request).

All placentas were described and sampled by generalist pathologists, who worked in a common network, where similar methods of storage and analysis, a similar form for requesting pathological analysis, and similar criteria for requesting exams were applied. Gross examination was performed after fixation in 10% formalin. Placental weight was determined 24 hours after delivery, trimmed, and fixed, and the percentile was determined according to placental weight charts (23). Five tissue samples were taken from each placenta for microscopic examination and were submitted for routine processing, paraffin embedding, and staining with hematoxylin and eosin.

Two expert placental pathologists performed the blinded histologic analysis. They were familiar with the methodology and nomenclature proposed in the Amsterdam Placental Workshop Group Consensus Statement (24) in 2014, according to which any placental abnormalities were named. Applying a classification model used by Redline in 2015 (25), six main classes of histologic lesion were recorded: (1) maternal vascular malperfusion (MVM), (2) fetal vascular malperfusion (FVM), (3) chorioamnionitis, (4) fetal inflammatory response (FIR), (5) abnormal umbilical insertion site (AUIS), and (6) meconium-associated changes (MAC).

The number of lesions for each category was recorded, ranging from zero (i.e., no lesions) to five.

### 2.1 Statistical analyses

We analyzed the distribution of maternal/pregnancy characteristics, neonatal clinical data, and placental lesion categories across the four groups. Categorical variables were summarized using absolute frequencies and proportions and the Chi-squared test was performed for statistical comparison. Continuous variables were described using medians and inter-quartile ranges (IQRs), and the Kruskal–Wallis test was applied.

### 2.2 Ethical approval

The study was approved by Institutional Review Board (San Gerardo Hospital’s Ethical Committee, 23rd December 2014, reference number 482).

## 3 Results

Neonatal characteristics are summarized in **Table 1**. As expected (19), a lower median UA pH was registered in the cases than in the controls. A similar trend was noted for UA BE. Apgar score at 5 minutes was lowest in encephalopathic infants.

**Table 1:** Neonatal characteristics of the study groups.

Characteristic		Group A	Group B	Group C	Group D	*p*-value
		*N*=173	*N*=32	*N*=50	*N*=68	
Place of birth	Central	33 (19.1)	20 (62.5)	0 (0.0)	68 (100.0)	<0.001
	Peripheral	140 (80.9)	12 (37.5)	50 (100.0)	0 (0.0)	
Male		96 (55.5)	19 (59.4)	20 (40.0)	33 (48.5)	0.191
Birth weight (g)		3,260 [3,000, 3,570]	3,150 [2,958, 3,463]	3,445 [3,153, 3,655]	3,110 [2,845, 3,425]	0.001
UA pH		7.04 [6.98, 7.11]	6.92 [6.82, 6.99]	7.24 [7.21, 7.29]	7.28 [7.19, 7.31]	<0.001
UA BE		−13.9 [−15.6, −12.9]	−16.4 [−19.1, −12.7]	−3.6 [−5.3, −1.4]	−5.3 [−7.5, −2.7]	<0.001
UA lactates		10.4 [9.0, 12.0]	10.5 [9.3, 13.7]	NA [NA, NA]	3.3 [2.0, 4.6]	<0.001
5-minute Apgar score		9 [8, 10]	4.50 [3, 7]	10 [10, 10]	10.00 [10, 10]	<0.001

Group A: asphyxiated newborns without hypoxic-ischemic encephalopathy.

Group B: asphyxiated newborns with hypoxic-ischemic encephalopathy.

Group C: non-asphyxiated newborns from low-risk pregnancies.

Group D: non-asphyxiated newborns from high-risk pregnancies.

UA, umbilical artery; BE, base excess.

Data are presented as median [IQR] or number (%).

Chi-square test for categorical variables, Kruskal–Wallis test for continuous variables.

Concerning maternal characteristics (**Table 2**), nulliparity was significantly (*p*<0.001) more frequent in the cases (84.3% of group A and 78% of group B) than in the controls (34% of group C and 63.2% of group D). BMI at delivery was significantly lower in group C than in the other groups (*p*<0.001). Additional data about the antenatal characteristics of the study groups are presented in **Supplementary Table 1**.

**Table 2:** Maternal characteristics of the study groups.

Characteristic	Group A	Group B	Group C	Group D	*p*-value
	*N*=173	*N*=32	*N*=50	*N*=68	
Age at delivery, years	33 [29, 37]	33 [30, 37]	33 [30, 35]	34 [31, 38]	0.444
Caucasian	155 (90.1)	25 (78.1)	43 (86.0)	55 (80.9)	0.131
Nulliparous	145 (84.3)	25 (78.1)	17 (34.0)	43 (63.2)	<0.001
Previous cesarean section	10 (5.8)	5 (15.6)	1 (2.0)	8 (11.8)	0.050
Assisted reproductive technique	6 (3.5)	2 (6.2)	0 (0.0)	6 (8.8)	0.105
BMI at delivery	28.3 [25.7, 31.0]	29.0 [26.4, 30.5]	25.0 [23.0, 28.0]	27.6 [24.8, 30.6]	<0.001

Group A: asphyxiated newborns without hypoxic-ischemic encephalopathy.

Group B: asphyxiated newborns with hypoxic-ischemic encephalopathy.

Group C: non-asphyxiated newborns from low-risk pregnancies.

Group D: non-asphyxiated newborns from high-risk pregnancies.

BMI, Body Mass Index.

Data are presented as median [IQR] or number (%).

Chi-square test for categorical variables, Kruskal–Wallis test for continuous variables.

Intrapartum data are presented in **Table 3**. Gestational age at delivery was significantly lower in group D than in the other groups (*p*<0.001). The majority of labors (92%) in the physiological group (group C) were spontaneous (*p*<0.001), while in the other groups labor was induced more often than in group C by a similar amount. The mode of delivery was different among the groups (*p*<0.001).

**Table 3:** Intrapartum characteristics of the study groups.

Characteristic		Group A	Group B	Group C	Group D	*p*-value
		*N*=173	*N*=32	*N*=50	*N*=68	
Gestational age at delivery, weeks		40.0 [39.0, 41.0]	40.0 [39.0, 40.0]	40.0 [39.0, 40.8]	39.0 [37.8, 39.0]	<0.001
Gestational age at delivery >40		44 (25.7)	6 (18.8)	13 (26.0)	6 (8.8)	0.029
Labor	Induced	65 (37.6)	14 (43.8)	4 (8.0)	35 (51.5)	<0.001
	No labor	3 (1.7)	6 (18.8)	0 (0.0)	9 (13.2)	
	Spontaneous	105 (60.7)	12 (37.5)	46 (92.0)	24 (35.3)	
Delivery	Spontaneous VD	93 (53.8)	12 (37.5)	49 (98.0)	36 (52.9)	<0.001
	Elective CS	4 (2.3)	1 (3.1)	0 (0.0)	8 (11.8)	
	Urgent CS in labor	36 (20.8)	9 (28.1)	0 (0.0)	13 (19.1)	
	Urgent CS before labor	0 (0.0)	5 (15.6)	0 (0.0)	1 (1.5)	
	Operative VD	40 (23.1)	5 (15.6)	1 (2.0)	10 (14.7)	
Thick meconium at delivery		47 (27.8)	7 (22.6)	0 (0.0)	7 (10.3)	<0.001
Abnormal FHR monitoring before labor		4 (2.4)	3 (10.0)	0 (0.0)	3 (4.4)	0.08
Abnormal FHR monitoring in labor (category II and III)		101 (60.5)	17 (54.8)	0 (0.0)	14 (20.6)	<0.001
Sentinel events		41 (23.7)	14 (43.8)	0 (0.0)	1 (1.5)	<0.001

Group A: asphyxiated newborns without hypoxic-ischemic encephalopathy.

Group B: asphyxiated newborns with hypoxic-ischemic encephalopathy.

Group C: non-asphyxiated newborns from low-risk pregnancies.

Group D: non-asphyxiated newborns from high-risk pregnancies.

VD, vaginal delivery; CS, cesarean section; FHR, fetal heart rate.

Data are presented as median [IQR] or number (%).

Chi-square test for categorical variables, Kruskal–Wallis test for continuous variables.

Fetal heart rate tracing during labor was more frequently (*p*<0.001) classed as category II and III in asphyxiated neonates (60.5% of group A and 54.8% of group B) than in group C (0%) or group D (20,.6%). Similarly, thick meconium at delivery was observed significantly more often (*p*<0.001) in the cases (27.8% of group A and 22.6% of group B) than in group C (0%) or group D (10.3%). Sentinel events were present almost exclusively in asphyxiated neonates (23.7% of group A and 43.8% of group B), compared with a prevalence of 0% in group C and 1.5% in group D (*p*<0.001). The distribution of sentinel events in the study groups is presented in **Supplementary Table 2**.

### 3.1 Placental results

Placental biometric and histologic characteristics are presented in **Tables 4**, **5**.

**Table 4:** Placental biometry.

Factors		Group A	Group B	Group C	Group D	*p*-value
		*N*=173	*N*=32	*N*=50	*N*=68	
Placental weight, g		500.00 [423.00, 570.25]	515.00 [411.50, 601.25]	445.00 [390.00, 507.00]	517.00 [450.00, 611.50]	<0.001
Percentile of placental weight for gestational age	<25th	43 (26.9)	10 (31.2)	24 (49.0)	10 (4.7)	<0.001
	>75th	37 (23.1)	9 (28.1)	2 (4.1)	25 (36.8)	
	25th–75th	80 (50.0)	13 (40.6)	23 (46.9)	33 (48.5)	
Umbilical cord length, cm (median [IQR])		35.00 [28.00, 47.50]	30.00 [25.00, 36.50]	35.00 [33.00, 40.00]	28.50 [24.00, 40.50]	0.004

Group A: asphyxiated newborns without hypoxic-ischemic encephalopathy.

Group B: asphyxiated newborns with hypoxic-ischemic encephalopathy.

Group C: non-asphyxiated newborns from low-risk pregnancies.

Group D: non-asphyxiated newborns from high-risk pregnancies.

Data are presented as median [IQR] or number (%).

Chi-square test for categorical variables, Kruskal–Wallis test for continuous variables.

**Table 5:** Placental histopathology characteristics.

	Level	Group A	Group B	Group C	Group D	*p*-value
		*N*=173	*N*=32	*N*=50	*N*=68	
Maternal vascular malperfusion	None lesion	33 (19.1)	4 (12.5)	1 (2.0)	12 (17.6)	<0.001
	1 lesions	75 (43.4)	16 (50.0)	16 (32.7)	23 (33.8)	
	2 lesions	49 (28.3)	6 (18.8)	15 (30.6)	20 (29.4)	
	3 lesions	16 (9.2)	6 (18.8)	9 (18.4)	11 (16.2)	
	4 lesions	0 (0.0)	0 (0.0)	7 (14.3)	2 (2.9)	
	5 lesions	0 (0.0)	0 (0.0)	1 (2.0)	0 (0.0)	
Fetal vascular malperfusion	None lesion	154 (89.0)	27 (84.4)	32 (65.3)	39 (57.4)	<0.001
	1 lesions	18 (10.4)	5 (15.6)	14 (28.6)	24 (35.3)	
	2 lesions	1 (0.6)	0 (0.0)	2 (4.1)	4 (5.9)	
	3 lesions	0 (0.0)	0 (0.0)	1 (2.0)	1 (1.5)	
Chorioamnionitis	No	144 (83.3)	23 (72)	40 (79.6)	53 (77.9)	Not significant for all
	Stage 1	12 (6.9)	2 (6.2)	6 (12.2)	1 (1.5)	
	Stage 2	14 (8.1)	5 (15.6)	4 (8.2)	10 (14.7)	
	Stage 3	3 (1.7)	2 (6.2)	0 (0.0)	4 (5.9)	
Fetal inflammatory response	No	156 (90.2)	27 (84.4)	42 (83,6)	58 (85.3)	Not significant for all
	Stage 1	5 (2.9)	2 (6.2)	4 (8.2)	3 (4.4)	
	Stage 2	11 (6.4)	3 (9.4)	4 (8.2)	7 (10.3)	
	Stage 3	1 (0.5)	0 (0.0)	0 (0.0)	0 (0.0)	
Abnormal umbilical insertion site	Yes	16 (9.9)	4 (12.9)	0 (0.0)	8 (11.7)	Not significant
	No	148 (90.1)	28 (87.1)	49 (100)	60 (88.3)	
Meconium-associated changes	Yes	12 (6.9)	2 (6.2)	0 (0.0)	0 (0.0)	0.039
	No	161 (93.1)	30 (93.8)	49 (100)	68 (100)	

Group A: asphyxiated newborns without hypoxic-ischemic encephalopathy.

Group B: asphyxiated newborns with hypoxic-ischemic encephalopathy.

Group C: non-asphyxiated newborns from low-risk pregnancies.

Group D: non-asphyxiated newborns from high-risk pregnancies.

Data are presented as number (%).

MVM, maternal vascular malperfusion; FVM, fetal vascular malperfusion; FIR, fetal inflammatory response; AUIS, abnormal umbilical insertion site; MAC, meconium-associated changes.

Chi-square test used.

The number of placentas with at least one lesion was very high in all groups (93.6% of group A, 100% of group B, 98% of group C, and 98.5% of group D).

Maternal vascular malperfusion (MVM) was significantly more frequent in group C than in the other groups (*p* = <0.001). Considering lesions of the maternal compartment, a greater number of concomitant vascular lesions was observed in the non-asphyxiated groups. No asphyxiated newborn (with or without HIE) had a high number (4 or 5) of MVM lesions. Fetal vascular malperfusion (FVM) was more frequent in non-asphyxiated newborns (34.7% of group C and 42.6% of group D) than in asphyxiated newborns (11% of group A and 15.6% of group B, *p* = <0.001). Fetal vascular lesions numbering ≥ 2 were mostly recorded in non-asphyxiated newborns. Only one case among the asphyxiated neonates had ≥ 2 concomitant fetal vascular lesions. Overall, no association between vascular placental lesions and neonatal asphyxia was observed. There was no statistically significant difference between groups in the prevalence of inflammatory lesions of the maternal and fetal compartment or in the abnormal umbilical insertion site.

Histologic meconium-associated changes (MAC) were observed in asphyxiated newborns only (6.9% of group A and 6.2% of group B, *p* = 0.039).

## 4 Discussion

Our study confirms the role that antepartum risk factors, such as nulliparity and obesity, and intrapartum events may have in causing and predicting neonatal asphyxia, as previously described in the literature (3–5). Moreover, sentinel events were documented almost exclusively in asphyxiated neonates and more frequently in those who developed HIE, thus further emphasizing the decisive role of acute catastrophic events (7, 9).

From the review of FHR tracing, the prevalence of abnormal FHR monitoring before or during labor in group A (62.9%) and in group B (64.8%) is in line with the previous findings by Clark et al, which indicated that approximately only half of infants who are born with metabolic acidemia could be identified with FHR monitoring (26). Furthermore, the prevalence of abnormal FHR monitoring before or during labor was also high in group D (25%). This underlines the limitations of electronic FHR monitoring in predicting perinatal asphyxia and the poor specificity of this test.

Regarding the placental results, we did not find in our study any association between vascular placental lesions and neonatal asphyxia. A unique aspect of our study is that we evaluated asphyxiated newborns without HIE, about which the availability of literature is poor. Avagliano et al. (27) in 2015 compared the placental pathology in 34 cases with severe fetal acidemia (cord pH at birth ≤7.0), 17.6% of which developed neonatal encephalopathy, with 102 controls (normal pH at birth) in low-risk term pregnancies. They observed that the following conditions affected cases significantly more frequently than controls: diffuse villous edema (38.2% vs. 0%, *p* < 0.0001), increased number of syncytial knots (41.2% vs. 13.7%, *p* = 0.002), and villous branching abnormalities (i.e., lesions that may be categorized as maternal vascular malperfusion) (35.2% vs. 14.7%, *p* = 0.02). It should be noted that the lesions reported in this study could be focal and our samples could have underestimated their prevalence.

In contrast to what was previously reported, we did not observe an association between vascular lesions and neonatal encephalopathy (10, 16, 17, 28). Recently, Vik et al. (18) conducted a retrospective case–control study that compared the placentas of infants that had neonatal encephalopathy with those of randomly selected control infants. No difference in maternal vascular malperfusion was found between cases and controls, while global FVM was three times more frequent in cases (20%) than in controls (7%) (*p* = 0.001). Notably, in this study, cases of neonatal encephalopathy were not only of hypoxic-ischemic origin. Interestingly, in our study, FVM was present at a similar frequency among cases (11% of group A and 15.6% of group B), but was more frequently among controls (34.7% of group C and 42.6% of group D). A possible explanation is that maternal and fetal malperfusion lesions were more common in Groups C and D as these lesions need time to develop (at least days), while the *in utero* asphyxia developed intrapartum in Groups A and B.

Bingham et al. (29) in their retrospective case–control study found that placental lesions were more frequent in neonates with moderate/severe HIE than in peer neonates admitted to the NICU for other reasons (87% vs. 65%, *p* = 0.008). Their controls were selected from newborns admitted to the NICU with a hospital stay less than 7 days and for whom placental sections were available. HIE newborns had more maternal vascular malperfusion (46% vs. 25%, *p* = 0.02), fetal vascular malperfusion (13% vs. 0%, *p* < 0.001), and clinical abruption (22% vs. 4%, *p* = 0.001). Our study differs to this owing to a similar presence of these histologic lesions in the controls, excluding abruption.

Interestingly, even if present in a small number of cases only, we observed placental histologic meconium-associated changes in asphyxiated newborns only (6.9% of group A and 6.2% of group B vs. 0% of groups C and D). This result is compatible with the greater prevalence of meconium-stained amniotic fluid observed in groups A and B and is in line with the correlation reported in the literature between meconium-stained amniotic fluid and low Apgar scores, an umbilical artery pH < 7.1, and hypoxic-ischemic encephalopathy (5, 30). Even if the presence of meconium-stained amniotic fluid is not pathological in most cases, the rate of complications when meconium is present is higher than with clear amniotic fluid (5, 31). Whether meconium contributes to fetal distress or is a manifestation of an underlying pathologic process remains controversial. A recent retrospective cohort study (32) compared singleton deliveries complicated by meconium stained amniotic fluid with acute vs. continuous meconium exposure-associated placental changes. The acute exposure group had a shorter interval between the rupture of membranes and delivery, and higher rates of non-reassuring FHR in labor and adverse neonatal outcomes. Unfortunately, in our study we could not establish the timing of exposure from the histologic specimens.

The role of chorioamnionitis as a risk factor of severe HIE is still debated in the literature. In term infants, some authors have found a significant association between chorioamnionitis and cerebral palsy, whereas others suggested a relation only with mild HIE (10, 33–35). Wintermark et al. (36) conducted a prospective cohort study of the placentas of term newborns with HIE admitted to the NICU who met the criteria for induced hypothermia; it was found that acute chorioamnionitis combined with fetal vasculitis and chorionic plate meconium was significantly associated with a greater degree of brain injury as observed by MRI. In a study by Frank et al. (37), the overall incidence of chorioamnionitis in newborns with HIE subjected to therapeutic hypothermia was 38%, compared with 18% in a cohort of healthy term infants. In our study, the prevalence of maternal and fetal inflammatory response was similar in all groups, suggesting that the role of inflammation, even if involved in brain damage, is not directly related, as expected, to the process of hypoxia-ischemia (4).

An important aspect of our study was the evaluation of placentas from controls. We chose to include two prospectively collected control groups of non-asphyxiated neonates: one from pathological pregnancies and one from well-defined low-risk pregnancies. Pathak et al. (38), in their prospective cohort study, detected normal histologic findings in 72.1% of pregnancies with normal outcomes, but also in 79.1%, 66.6%, 80%, and 74.8% of pregnancies affected by pre-eclampsia, pregnancy-induced hypertension, gestational diabetes, and a small size of baby for the gestational age, respectively. In addition, they found that certain placental histologic findings were more common in particular outcome groups, but the overall sensitivity and positive predictive value for any specific type of histologic lesion were low. By contrast, in our study, the number of placentae with at least one kind of lesion was consistently high in all groups. Our results are similar to those of Romero et al. (39), who in a retrospective cohort study of placental samples from women with a singleton gestation delivered at term without obstetrical complications found that most placentas (78%) had some type of lesion (either inflammatory or vascular), but most were mild. All these results support the conclusion that in the context of an unselected population delivering at or near term, the clinical significance of many histologically detectable placental lesions remains uncertain, if not interpreted as part of the overall clinical context, as also recently argued by Polnaszek et al. (40).

Placental examination is an important diagnostic tool in assessing the etiology of asphyxia and HIE, and placental pathology might help to investigate the underlying timing of injury pathways in HIE and explain individual neonatal responses. A recent paper by Redline et al. (41) can reconcile the discrepancy between our results and previous literature and contribute to interpreting our findings. They demonstrated that the inter-observer reliability for placental lesions defined by the Amsterdam Classification is good to excellent in the evaluation of inflammatory lesions but poor for vascular lesions, especially those of the maternal compartment. The authors stressed the need for further refinement of the classification and better educational efforts to fully implement the Amsterdam system. This would allow the results of different studies to be compared with greater reliability, possibly distinguishing between chronic and recent lesions (42).

### 4.1 Strengths and limitations

Strengths of the study were the well-defined distinction between the four groups of newborns, the large number of asphyxiated neonates included, the prospective multicenter collection of controls among newborns from low-risk pregnancies, and the standardized blinded examination of placental specimens by a placental pathology expert.

A limitation of the study was the poor number of asphyxiated newborns with diagnosis of HIE, due to the low prevalence of this condition. Other studies with adequate groups of controls are necessary to better understand the role of the placenta in neonatal hypoxic-ischemic damage. Another limitation was that, unfortunately, a some cases in the cohort did not receive a placental examination, even when it was recommended that all centers send the placentas of asphyxiated neonates for examination. Moreover, placentas from group A and B were sampled by generalist pathologists, who may have less closely adhered to the Amsterdam Statement. This also limited our ability to optimally evaluate placental biometry.

## 5 Conclusion

When asphyxia or HIE occurs, it is useful for the clinician to know if the event was foreseeable and if knowledge of the risk factors could have led them to manage the case differently. It is also useful to know if antecedents such as placental characteristics could have been a preconditioning factor for the observed events. Our study confirms that asphyxia occurs in the presence of some risk factors and especially that it can be preceded by acute events. However, the role of placental lesions as determined using the traditional analysis and classification methods, like other unexplored factors such as genetic or environmental factors, remains uncertain and therefore requires further investigation.

## Data availability statement

The raw data supporting the conclusions of this article will be made available by the authors, without undue reservation.

## Ethics statement

The studies involving humans were approved by San Gerardo Hospital’s Ethical Committee, 23rd December 2014, reference number 482. The studies were conducted in accordance with the local legislation and institutional requirements. The participants provided their written informed consent to participate in this study.

## Author contributions

All authors contributed to the study conception and design. Protocol development was carried out by GP, SM and AL. Material preparation, data collection, and data management were performed by SA, LL, FM, VB, and MC. Data analysis was performed by DB, SA, LL, and AL. The first draft of the manuscript was written by SA, LL, FM, and VB, and edited by AL and FM. All authors contributed to the article and approved the submitted version.
