# Supplementary material for: Placental pathology in perinatal asphyxia: a case–control study
Source: Front Clin Diabetes Healthc. 2023 Sep 18;4:1186362. doi: 10.3389/fcdhc.2023.1186362 (PMC10545088; doi:10.3389/fcdhc.2023.1186362)
Supplement: Supplementary file 1 [file Table_1.docx]

**Table 1. Antenatal characteristics of the study groups**

| Factors | Group A | Group B | Group C | Group D | p value |
| --- | --- | --- | --- | --- | --- |
|  | N=173 | N=32 | N=50 | N=68 |  |
| CHRONIC HYPERTENSION | 1 (0.6) | 0 (0.0) | 0 (0.0) | 3 (4.4) | 0.065 |
| GESTATIONAL HYPERTENSION | 9 (5.2) | 6 (18.8) | 0 (0.0) | 12 (17.6) | <0.001 |
| PREECLAMPSIA | 7 (4.1) | 3 (9.4) | 0 (0.0) | 7 (10.3) | 0.051 |
| POSITIVE THROMBOPHILIC STATUS | 3 (1.7) | 1 (3.1) | 0 (0.0) | 2 (2.9) | 0.643 |
| GESTATIONAL DIABETES | 18 (10.4) | 4 (12.5) | 1 (2.0) | 14 (20.6) | 0.017 |
| PREGESTATIONAL DIABETES | 4 (2.3) | 0 (0.0) | 0 (0.0) | 4 (5.9) | 0.148 |
| PREGESTATIONAL HYPOTHYROIDISM | 4 (2.3) | 3 (9.4) | 0 (0.0) | 1 (1.5) | 0.049 |
| GESTATIONAL HYPOTHYROIDISM | 4 (2.3) | 0 (0.0) | 2 (4.0) | 4 (5.9) | 0.355 |
| HYPERTHYROIDISM | 1 (0.6) | 0 (0.0) | 0 (0.0) | 0 (0.0) | 0.832 |
| GESTATIONAL CHOLESTASIS | 3 (1.7) | 0 (0.0) | 0 (0.0) | 10 (14.7) | <0.001 |
| ANTEPARTUM HAEMORRHAGE | 0 (0.0) | 1 (3.1) | 2 (4.0) | 2 (2.9) | 0.109 |
| INTRAPARTUM FEVER | 13 (7.6) | 0 (0.0) | 0 (0.0) | 9 (13.2) | 0.015 |
| CHORIOAMNIONITIS | 6 (3.5) | 0 (0.0) | 0 (0.0) | 4 (5.9) | 0.216 |
| OLIGOHYDRAMNIOS | 10 (5.8) | 1 (3.1) | 0 (0.0) | 1 (1.5) | 0.17 |
| POLYHYDRAMNIOS | 9 (5.2) | 4 ( 12.5) | 0 (0.0) | 1 (1.5) | 0.028 |
| FETAL GROWTH RESTRICTION | 6 (3.5) | 0 (0.0) | 0 (0.0) | 6 (8.8) | 0.043 |

Group A: asphyxiated newborns without hypoxic- ischemic encephalopathy

Group B: asphyxiated newborns with hypoxic- ischemic encephalopathy

Group C: non-asphyxiated newborns from physiological pregnancies

Group D: non-asphyxiated newborns from at risk pregnancies

Data are presented as number (%)

* Chi-square test for categorical variables, Kruskal-Wallis test for continuous variables

**Table 2. Sentinel events**

| Factors | Group A | Group B | Group C | Group D | P value |
| --- | --- | --- | --- | --- | --- |
|  | N=173 | N=32 | N=50 | N=68 |  |
| FETAL BRADYCARDIA | 31 (18.2) | 6 (18.8) | 0 (0.0) | 0 (0) | <0.001 |
| PLACENTAL ABRUPTIO | 3 (1.7) | 4 (12.5) | 0 ( 0.0) | 0 (0) | <0.001 |
| UTERINE RUPTURE | 0 (0) | 2 (6.2) | 0 (0.0) | 1 (1.5) | 0.007 |
| SHOULDER DYSTOCIA | 5 (2.9) | 3 (9.4) | 0 (0.0) | 1 (1.5) | 0.072 |
| CORD PROLAPSE | 1 (0.6) | 0 ( 0.0) | 0 (0.0) | 0 ( 0.0) | 0.834 |
| AMNIOTIC FLUID EMBOLISM | 1 (0.6) | 0 ( 0.0) | 0 (0.0) | 0 ( 0.0) | 0.833 |
| ≥ 1 SENTINEL EVENT | 41 (23.7) | 14 (43.8) | 0 (.0) | 1 (1.5) | <0.001 |

Group A: asphyxiated newborns without hypoxic- ischemic encephalopathy

Group B: asphyxiated newborns with hypoxic- ischemic encephalopathy

Group C: non-asphyxiated newborns from physiological pregnancies

Group D: non-asphyxiated newborns from at risk pregnancies

Data are presented as number (%)

* Chi-square test for categorical variables, Kruskal-Wallis test for continuous variables
